# Zero-Watermarking for Vector Maps Combining Spatial and Frequency Domain Based on Constrained Delaunay Triangulation Network and Discrete Fourier Transform

**DOI:** 10.3390/e25040682

**Published:** 2023-04-19

**Authors:** Xu Xi, Yang Hua, Yi Chen, Qiande Zhu

**Affiliations:** 1 School of Geography Science and Geomatics Engineering, Suzhou University of Science and Technology, Suzhou 215000, China; 2 State Key Laboratory of Hydrology and Water Resources and Hydraulic Engineering Science, Nanjing Hydraulic Research Institute, Nanjing 210029, China

**Keywords:** zero-watermarking, vector maps, constraint Delaunay triangulation networks, discrete Fourier transform

## Abstract

With its lossless properties, zero-watermarking has attracted a lot of attention in the field of copyright protection for vector maps. However, the common zero-watermarking algorithm puts too much emphasis on mining for global features, making it vulnerable to cropping attacks, and the robustness is not comprehensive enough. This study provides a vector map zero-watermarking scheme that utilizes spatial statistical information and frequency domain transformation methods in an effort to solve the aforementioned issue. In order to make the scheme more resistant to cropping and compression, it is constructed on the basis of feature point extraction and point constraint blocking of the original vector map. Within each sub-block, feature points are used to build constraint Delaunay triangulation networks (CDTN), and the angular values within the triangle networks are then extracted as spatial statistics. The angle value sequence is further transformed by discrete Fourier transform (DFT), and the binarized phase sequence is used as the final feature information to build a zero watermark by executing an exclusive disjunction operation with the encrypted copyright watermark image, both of which contribute to the scheme’s robustness and security. The results of the attack experiments show that the proposed vector map zero-watermarking can restore identifiable copyright images under common geometric attacks, cropping attacks, and coordinate system transformations, demonstrating a high level of robustness. The theoretical basis for the robustness of this watermarking scheme is the stability of CDTN and the geometric invariance of DFT coefficients, and both theory and experiment validate the method’s validity.

## 1. Introduction

With the characteristics of precise positioning, high accuracy, small storage capacity, and broad application, vector maps play a crucial and fundamental role in the construction of numerous fields, such as smart cities, national defense deployment, ecological protection, new agriculture, and disaster prevention and control, thereby establishing their extremely high commercial value [1,2,3]. Now more than ever, producers, legal owners, and consumers of vector maps are suffering enormous losses as a result of unlawful trafficking, dissemination, alteration, and unauthorized use of the data because of the ease with which electronic data may be transmitted. Therefore, there is a huge contradiction between the sharing and security of vector map data, and its security protection is highly valued by governments and scholars [4,5,6].

As the cutting-edge technique for copyright protection of digital products, digital watermarking provides technological support for the security protection of vector maps and the healthy growth of the geographic information industry; thus, it has been a subject of considerable interest [7,8,9,10]. Traditional digital watermarking conceals copyright information in electronic data invisibly using an algorithm that does not compromise data quality or availability. In addition, the invisible nature of the watermark ensures that other data consumers are unaware of its presence, hence enhancing the security of watermarking. The traditional watermarking for vector maps is commonly accomplished by altering the coordinate values. The way is typically inapplicable to vector maps, which have extremely low redundancy and require very high accuracy in the use of data, particularly for certain high-precision data where even the tiniest deviation is undesirable. Therefore, it is crucial to building a lossless watermarking technique for vector map copyright protection. Zero-watermarking extracts the feature information of cover data to generate watermark keys as the basis for copyright verification, and its non-embedding mechanism has garnered significant interest [11,12,13]. The robustness and security of the algorithm depend heavily on the stability of extracted feature information. In vector map zero-watermarking, therefore, mining as much stable feature information as possible has been an essential study direction.

In this study, the benefits and drawbacks of spatial and frequency domain watermarking for vector maps are analyzed, and an attempt is made to extract the deep inherent feature information of vector maps by merging the spatial and frequency domains. On this basis, a robust zero-watermarking algorithm for vector maps is proposed. The contributions of this paper include the following:

(1) Analysis of the existing works from the perspectives of the spatial and frequency domains, respectively, leads to the conclusion that the relevant study is characterized by unicity and completeness in feature information mining, with relatively one-sided robustness.

(2) A vector map zero-watermarking combining spatial and frequency domains is proposed. In the spatial domain, a constrained Delaunay triangulation network (CDTN) is constructed after feature points and data blocking, and the angle values in CDTN are extracted as the initial feature information. In the frequency domain, the discrete Fourier transform (DFT) is used to further transform and mine sequences of angle values. The sequence of DFT phase coefficients in each sub-block is used as the final feature information for the generation of zero watermarks.

(3) Under the above operation, a zero-watermarking scheme for vector maps is built. Experimental results and analysis demonstrate that watermarking has a comprehensive robustness to common geometric attacks, coordinate point attacks, and clipping attacks, among others.

The rest of this paper is organized as follows: The related work is introduced in Section 2. The methodology containing basic algorithms and a detailed watermarking process is proposed in Section 3. Experimental results, analysis, and comparative works are provided in Section 4. Finally, some conclusions are given in Section 5.

## 2. Related Works

The vector map watermarking algorithm was first proposed and designed by Cox and Jager [14], who encoded watermark information directly on the coordinate values of each vertex. The method is also the first spatial domain watermarking for vector maps, but it is vulnerable to various simple attacks and is poorly concealed. In order to improve concealment and security, most vector map watermarking in the existing literature on a spatial domain would use the geometric statistical characteristics as the watermark embedding domain, including topological relations [15], angle values between feature vertices [16], element perimeters and areas [17], the spatial distance between different elements [18], etc. This collection of spatial statistical eigenvalues possesses a wide range of geometric invariants that can serve as a solid foundation for the applicable watermarking methods. However, all of these watermarking techniques accomplish watermark embedding by directly altering the coordinate values, which leaves them vulnerable to precision bit erasure and low robustness [19].

In contrast to the spatial domain watermarking method of directly manipulating coordinate values, the frequency domain watermarking of vector maps converts the coordinate domain to the frequency domain and inserts watermark information by adjusting the frequency coefficient [19]. Typical frequency transform methods include discrete Fourier transform (DFT), discrete wavelet transform (DWT), discrete cosine transform (DCT), and singular value decomposition (SVD) [19,20,21,22]. The transformation coefficients of these methods all provide a certain degree of geometric invariance, hence ensuring the robustness of related watermarking. Both spatial domain watermarking and frequency domain watermarking embed watermarks by modifying vertex coordinates. Many research used deliberate interference reduction strategies, but the quality and availability of watermarked vector maps are still affected to varying degrees. In this case, the zero-watermarking algorithm with lossless characteristics has attracted more and more attention [20,23,24,25].

Rather than altering the protected electronic data in any way of embedded watermarking, zero-watermarking extracts feature information from it to build the watermark and then utilizes the watermark pictures themselves as the secret key for copyright verification, with the help of a trusted third party [26]. The non-embedded way subverts the functional mechanism of conventional watermarking and achieves no interference with data in a true sense, which is ideal for the precise usage requirements of vector maps [27]. Depending on the type of feature information, vector map zero watermarking may be broken down into two categories: spatial domain statistical features-based zero watermarking and improved frequency domain-based zero watermarking.

In spatial statistical features-based zero watermarking, Sun et al. [28] built zero-watermarking by taking the distance between feature points and cluster centers in cluster blocks as feature information. The method is resistant to data reduction or compression and geometric modification. Wang et al. [29] divided the vector map into multiple rings with concentric circles, calculated the number of vertices in each ring as feature information, and constructed zero-watermarking images after the XOR operation with copyright information. This algorithm can effectively resist common geometric attacks and coordinate point attacks. Peng et al. [30] employed the distance ratio between each feature point and the geometric center of all feature points as the feature information to construct zero watermarking, and this method can better resist geometric attacks, interpolation, and simplification attacks.

Regarding the research of improved frequency domain zero watermarking, Lv et al. [31] performed DFT on the feature points and converted the phase value of DFT to the binary matrix as feature information to construct zero watermarks, and the scheme utilized the geometric invariance of DFT coefficients to demonstrate good robustness against translation, scaling, and points attacks. Li et al. [32] performed DFT on all coordinate values, converted the phase of DFT from radian values to angle values, selected uniformly distributed angle values to be rounded and converted into binary values, and then constructed a binary matrix as feature information for the zero watermark, which also inherits the advantages of DFT and shows good robustness in format conversion and projection transformation. Han [33] extracted the feature points and then decomposed them by DWT. The zero watermark was produced using the low-frequency coefficients of DWT, achieving robustness in anti-simplification, translation, scaling, etc.

Based on the aforementioned studies, we can conclude that the present research on zero watermarking of vector maps mostly consists of the following characteristics: (1) improved frequency domain-based zero watermarking inherits the advantage of geometric invariance of frequency coefficients, but in reality, it is also a specific statistical feature zero watermarking, with frequency domain coefficients serving as the statistical feature information; (2) the extracted feature information of vector maps has the overall characteristics, and a data can only obtain one zero watermark. In this case, the robustness of the scheme is often one-sided, and it is difficult to resist cropping attacks. In order to address this issue, this study seeks to integrate the stability properties of spatial domain statistics and frequency domain transformation coefficients with the block processing and feature points extraction in order to develop an all-sided robustness zero-watermarking for vector maps.

## 3. Methodology

In this section, we primarily introduce the preprocessing method for the original vector maps, the statistical information mining method in the spatial domain based on the constrained Delaunay triangulation network (CDTN), the feature information construction method based on DFT, and the specific implementation steps of zero watermark construction and extraction of the proposed watermarking.

### 3.1. Data Preprocessing

The data preprocessing session of the proposed watermarking mainly contains feature point extraction and data blocking. Feature point extraction refers to the extraction of some of the most stable vertices in a vector map by a specific algorithm, and feature points are required to be the key nodes that make up the vector map. If these feature points are corrupted, the data quality will be drastically reduced. This property protects the data and merely provides a theoretical foundation for the security and robustness of the zero watermarking scheme based on feature points. The Douglas–Peuker algorithm [34], which is a common way to simplify polylines, was used in this research to extract feature points. The larger the threshold value set for the Douglas–Peuker algorithm in the feature point extraction process, the fewer feature points are obtained, and the less information is used to construct the zero watermark, but the watermarking scheme is more resistant to compression attacks as well as coordinate point attacks. However, collecting fewer feature points results in fewer data being used to generate the zero watermark. Consequently, a suitable threshold value must be selected via trial and error prior to watermark construction to guarantee there are adequate and stable feature points for zero watermark creation.

Embedding watermarks in sub-blocks is a crucial method of the watermarking scheme against cropping attacks, as data blocking is to divide the original data into a number of sub-blocks, which is similar to cropping attacks. In this study, we chose the point-constrained blocking method to block the original vector maps, which is based on the average area blocking method, and the blocks with a number of points greater than the threshold continued to be divided until the number of points was less than the threshold [35]. The initial blocking area a and the threshold points count t must be adjusted accordingly. After reasonable debugging, the zero watermarks constructed on this basis were more uniformly and reasonably distributed in the data, as the number of points within each block was similar and the area difference was as small as possible.

### 3.2. Spatial Domain Statistical Information Mining Based on CDTN

The CDTN is made up of a series of connected but non-overlapping triangles and the outer circles of these triangles do not contain any other points in the domain, which is known as the empty outer circle property of CDTN [36]. Another property of CDTN is that the minimum angles of all triangles are maximized as much as possible, which indicates that all the triangles in CDTN are closest to regularization [36,37]. The above properties ensure that the construction of CDTN will form unique and reasonable triangles. Furthermore, adding, deleting, or moving some vertices affects only the adjacent triangle and has no effect on the construction of other triangles. As a result, CDTN has good global stability, which can serve as the foundation for the robust performance of watermarking.

In the proposed watermarking scheme, angle values in the CDTN are extracted as stable spatial statistics of vector maps and used for constructing feature information. The main steps are as follows:

(1) Construction of CDTN based on feature points. After the Douglas–Peuker simplification of the original vector map, obtain the discrete points set $V=\left\{v_{0},v_{1},v_{2}\;\ldots \;v_{i},\;\ldots \;v_{n}\right\}$, and randomly sort each point. If the point $v_{i}$ is inside a triangle, connect it to the triangle’s three vertices and divide the triangle into three triangles. If $v_{i}$ is on the side of a triangle, connect the disjoint points of the two triangles that share a side with $v_{i}$ and divide the two triangles that share a side into four triangles. The CDTN is formed after any vertex of the initial triangle is eliminated by regularizing the triangular dissection using the edge-swapping principle.

(2) Extraction of angle values. Generating triangle networks based on each block’s feature points, extracting angle values with the inverse trigonometric function (Equation (2)) by calculating the distance between each triangle (Equation (1)), and composing a sequence of angle values $A_{r}$ (Equation (3)), ensuring that the length of the angle sequence $A_{r}$ within each block is greater than the length of the watermark sequence $W_{inf}$.

(1) $$\left\{\begin{array}{c} d_{12}=\sqrt{{\left(v_{1}^{x}-v_{2}^{x}\right)}^{2}+{\left(v_{1}^{y}-v_{2}^{y}\right)}^{2}}\\ d_{23}=\sqrt{{\left(v_{2}^{x}-v_{3}^{y}\right)}^{2}+{\left(v_{2}^{x}-v_{3}^{y}\right)}^{2}}\\ d_{31}=\sqrt{{\left(v_{3}^{x}-v_{1}^{x}\right)}^{2}+{\left(v_{3}^{y}-v_{1}^{y}\right)}^{2}} \end{array}\right.$$

(2) $$\left\{\begin{array}{l} \alpha_{1}=arccos\;[(d_{12}^{2}+d_{31}^{2}-d_{23}^{2})/\left(2\cdot d_{12}\cdot d_{31}\right)]\\ \alpha_{2}=arccos\;[(d_{23}^{2}+d_{12}^{2}-d_{31}^{2})/(2\cdot d_{23}\cdot d_{12})]\\ \alpha_{3}=arccos\;[(D_{23}^{2}+D_{31}^{2}-D_{12}^{2})/(2\cdot D_{23}\cdot D_{31})] \end{array}\right.$$

(3) $$A_{r}=\left\{\alpha_{1},\alpha_{2},\alpha_{3},\cdot \cdots \alpha_{n}\right\},\;\;r=1,2,\ldots ,m$$

**Figure 1:**
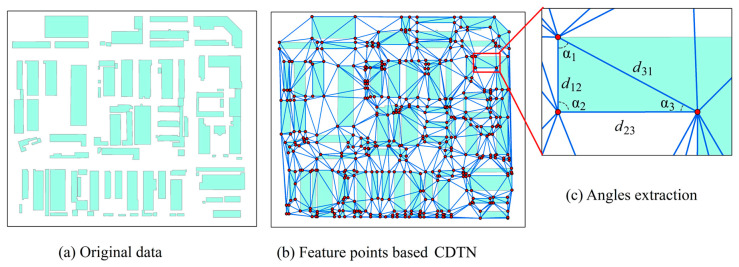
Extraction of angle values of CDTN.

In Equation (1), $v_{1}^{x}$, $v_{2}^{x}$, and $v_{3}^{x}$ are the horizontal coordinate values of the first, second, and third points in Figure 1, respectively; $v_{1}^{y}$, $v_{2}^{y}$, and $v_{3}^{y}$ are the vertical coordinate values of the first, second, and third points in Figure 1, respectively; and $d_{12}$, $d_{23}$, and $d_{31}$ are the distances between different vertices, respectively. In Equation (2), $\alpha_{1}$,$\alpha_{2}$, and $\alpha_{3}$ are the three interior angle values within a triangle in Figure 1. The $A_{r}$ denotes the set of angle values of the r-th block, m demotes the total number of sub-blocks, and n denotes the total number of angles in Equation (3). The angle sequence $A_{r}$ is based on CDTN’s invariance in the face of geometric attacks such as rotation, translation, and scaling. Based on this, we further processed $A_{r}$ to extract more robust information for the construction of zero-watermarking.

### 3.3. Feature Information Construction Based on DFT

The DFT is a fundamental Fourier analysis technique that converts signals from the time domain to the frequency domain [38]. DFT plays an important role in vector map watermarking schemes as a common method for frequency domain watermarking. The positive and inverse transform equations of DFT are shown in Equations (4) and (5), respectively.

(4) $$Y\left(k\right)=\sum_{n=0}^{N-1}x\left(n\right){\left(e^{-i2\pi ∕N}\right)}^{kn},\;k\in \left[0,N-1\right]$$

(5) $$x\left(n\right)=\frac{1}{N}\sum_{n=0}^{N-1}Y\left(k\right){\left(e^{i2\pi ∕N}\right)}^{kn},\;n\in \left[0,N-1\right]$$

where e denotes the natural logarithmic base, and i denotes the imaginary unit. The DFT is used to transform the angular values in CDTN and obtain the phase sequence as feature information, and the specific steps are shown as follows: by following the steps in Section 3.2 for obtaining the Angle values in the CDTN, the maximum and minimum values of each triangle Angle value are chosen to form the Angle sequence set $A=\left\{A_{1},A_{2},\ldots ,A_{m}\right\}$.

(6) $$\left\{\begin{array}{c} \alpha_{i}^{min}=min\{\alpha_{i}^{1},\alpha_{i}^{2},\alpha_{i}^{3}\}\\ \alpha_{i}^{max}=max\{\alpha_{i}^{1},\alpha_{i}^{2},\alpha_{i}^{3}\} \end{array}\right.$$

(7) $$A_{r}=\{\alpha_{1}^{min},\alpha_{1}^{max},\alpha_{2}^{min},\alpha_{2}^{max}\cdots \alpha_{k}^{min},\alpha_{k}^{min}\}$$

where $\alpha_{i}^{1},\alpha_{i}^{2}$, and $\alpha_{i}^{3}$ are the three interior angles of the *i*-th triangle; $\alpha_{i}^{min}$ and $\alpha_{i}^{max}$ are the minimum and maximum values of these three angular values, respectively. $A_{r}$ is the sequence of angle values of the *r*-th block in Equation (7), and *k* is the number of triangles of the *r*-th block. The complex number $A_{r}^{u}$ of the angle value is constructed by Equation (5). According to Equation (8), the discrete Fourier transform of complex sequence $\left\{A_{r}^{u}\right\}$ was performed, and the Fourier coefficient $\left\{A_{r}^{v}\right\}$ was obtained (Equation (9)). According to the property of Fourier coefficients, the amplitude sequence $R_{r}^{v}$ and phase value sequence $P_{r}^{v}$ of the *r*-th block can be obtained through Equations (10) and (11), in which $P_{r}^{v}$ is represented by angle value.

(8) $$A_{r}^{u}={\left(\alpha_{min}\right)}_{u}+i\cdot {\left(\alpha_{max}\right)}_{u},\left(u=0,1,2,3\ldots k-1\right)$$

(9) $$A_{r}^{v}=\sum_{u=0}^{k-1}A_{r}^{u}{\left(e^{-i2\pi /k}\right)}^{uv},\left(v=0,1,2,3\ldots k-1\right)$$

(10) $$R_{r}^{v}=\left|A_{r}^{u}\right|=\sqrt{{\left[R\left(A_{r}^{v}\right)\right]}^{2}+{\left[I\left(A_{r}^{v}\right)\right]}^{2}},R_{r}=\left\{R_{r}^{v}\right\}$$

(11) Prv=arctanRArvIArv,Prv∈0,180,Pr=Prv

where *u* is the number of triangles in $A_{r}$, and *i* is the imaginary unit of complex numbers in Equation (8); *v* is the DFT series in Equation (9); and $R\left(A_{r}^{v}\right)$ and $I\left(A_{r}^{v}\right)$ represent the real and imaginary parts of the Fourier coefficients $A_{r}^{u}$, respectively. Then, each angle value in the $P_{r}^{v}$ is rounded down and converted into 8-bit binary form to form the feature sequence set $P_{r}^{binary}$. The lengths of each sequence in $P_{r}^{binary}$ are compared with the watermark sequence $W_{inf}$, and the supplementary bits strategy in Equation (12) is adopted to make $P_{r}^{binary}$ equal to the length of the watermark information $W_{inf}$. To improve watermarking security, $W_{inf}$ is scrambled, yielding the encrypted watermark information sequence $W_{inf}^{ecy}$. Finally, Xor operations are performed on $P_{r}^{binary}$ and $W_{inf}^{ecy}$, and each block obtains a zero watermark $W_{r}^{zero}$ (Equation (13)).

(12) $$\left\{\begin{array}{l} e=\frac{L\left(W_{inf}\right)}{L\left(P_{r}^{binary}\right)}\\ P_{r}^{binary}=P_{r}^{binary}\ll \left(e-1\right)P_{r}^{binary} \end{array}\right.$$

(13) $$W_{r}^{zero}=P_{r}^{binary}⊕W_{inf}^{ecy},r=1,2\ldots m$$

### 3.4. Procedure of the Proposed Zero-Watermarking

**Figure 2:**
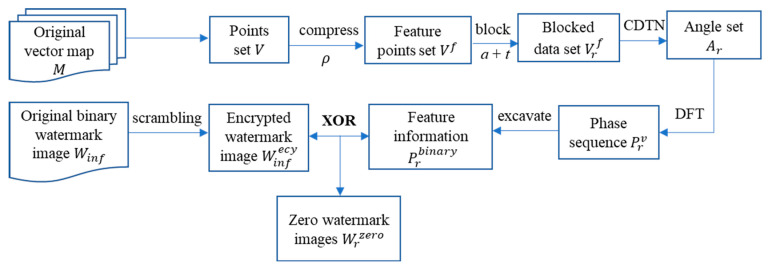
Procedure of the proposed watermarking scheme.

Based on the aforementioned methods, the specific process of the proposed vector map zero-watermarking is depicted in Figure 2, with the following steps:

*Step* 1: Firstly, the Douglas–Peucker algorithm is used to compress the original vector map M by setting the compression threshold as *ρ* and obtaining the feature points set $V^{f}$. The point-constrained blocking method is then used to block the feature point set $V^{f}$, with the setting of the initial blocking area as a and the threshold number of points as *t* to divide the data into m sub-blocks, and each sub-block can obtain a feature point set $V_{r}^{f}$.

*Step* 2: Every sub-block can create a CDTN with feature points $V_{r}^{f}$ and Equations (1) and (2) are used to calculate the angles of all triangles in the CDTNs. The maximum and minimum angles of each triangle are then selected to compose an angular sequence set $A_{r}$.

*Step* 3: Equation (9) is used to build the complex sequence of angle values $A_{r}^{v}$, and Equation (10) is used to obtain the sequence of phase values $P_{r}^{v}$ following DFT. To create the feature sequence set $P_{r}^{binary}$, each angle value in the sequence $P_{r}^{v}$ is rounded down and converted to an 8-bit binary representation. $P_{r}^{binary}$ is made equal length, with $W_{inf}$ in accordance with the supplementary bits strategy in Equation (12).

*Step* 4: The binary image containing copyright content is taken as the initial watermark image W, and the binary watermark sequence $W_{inf}$ is read. The Arnold scrambling algorithm is employed to encrypt the watermark image, yielding the disordered watermark sequence $W_{inf}^{ecy}$.

*Step* 5: The Xor operation between $P_{r}^{binary}$ and $W_{inf}^{ecy}$ yields one zero-watermark $W_{r}^{zero}$ for each block and m distinct zero-watermark images in total. All zero-watermark images and the original watermark images are submitted to the intellectual property rights (IPR) center for preservation, and time stamps are added to resist interpretation attacks.

### 3.5. Watermark Detection

**Figure 3:**
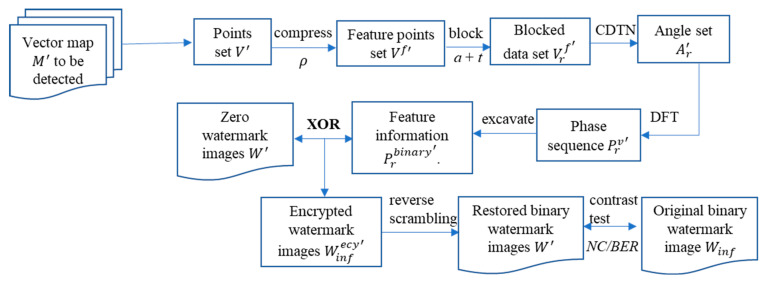
Procedure of watermark extraction and copyright authentication.

Watermark detection is the inverse process of watermark creation, and the settings of each execution step must be compatible with the parameters established during the construction process, the specific process is shown in Figure 3. The feature points set $V^{\prime }$ is obtained by applying the same degree of Douglas–Peucker compression to the vector map to be detected, then performing a block operation on $V^{\prime }$ with the same parameters as when constructing the zero watermark, and constructing a CDTN within each sub-block, recording the maximum and minimum interior angles of each triangle to form a sequence of angle values $A_{r}^{\prime }$. According to Equations (8) and (9), the complex sequence of angle values ${A_{r}^{u}}^{\prime }$ of each sub-block $A_{r}^{\prime }$ are constructed, DFT is applied to acquire the Fourier coefficient sequence ${A_{r}^{v}}^{\prime }$, and then the phase value sequence ${P_{r}^{v}}^{\prime }$ of each sub-block is obtained. The angular values in ${P_{r}^{v}}^{\prime }$ are rounded down and converted to binary to form the feature sequence set ${P_{r}^{binary}}^{\prime }$.

All zero-watermark images $W_{r}^{zero}$ are obtained from the IPR matrix $W_{r}^{zero}$ and matched with the elements in ${P_{r}^{binary}}^{\prime }$. The Xor operation is then applied to the successfully matched sequence to obtain multiple encrypted watermark images ${W_{inf}^{ecy}}^{\prime }$ (Equation (14)).

(14) $${W_{inf}^{ecy}}^{\prime }={P_{r}^{binary}}^{\prime }⊕W_{r}^{zero}$$

The inverted Arnold scrambling is employed on an encrypted watermark image ${W_{inf}^{ecy}}^{\prime }$ to obtain the decrypted watermark image $W^{\prime }$, and then $W^{\prime }$ is compared with the original watermark image W for verification. Normalized Correlation (NC) is used to measure the similarity between images [3] (Equation (15)).

(15) $$NC=\frac{\sum_{i,j}W_{i,j}*W_{i,j}^{\prime }}{\sqrt{\sum_{i,j}W_{i,j}^{2}}\sqrt{\sum_{i,j}W_{i,j}^{{}^{\prime }2}}}$$

where $W_{i,j}$ and $W_{i,j}^{\prime }$ are original and extracted watermark bit information at the coordinates of (*i, j*), respectively. Bit error rate (BER) is used to evaluate the specific error size of the extracted watermarks [3] (Equation (16)).

(16) $$BER=\frac{100}{l}\sum_{i,j=0}^{l-1}\left\{\begin{array}{c} 0\;W_{i,j}=W_{i,j}^{\prime }\\ 1\;W_{i,j}\neq W_{i,j}^{\prime } \end{array}\right.$$

where l denotes the length of watermark information.

## 4. Results and Discussion

### 4.1. Experimental Results

#### 4.1.1. Watermark Encryption

**Figure 4:**
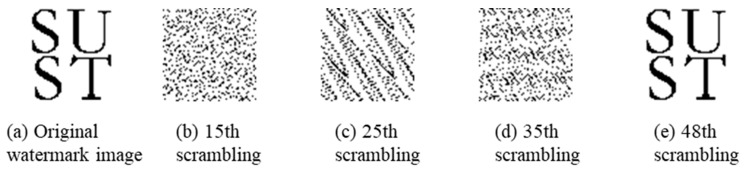
Watermark image scrambling.

**Figure 5:**
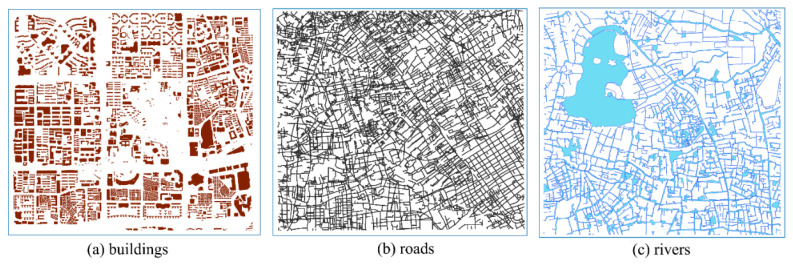
Original vector maps.

The watermark image utilized in this paper to create zero watermarks is a binary image with a size of 64 × 64 pixels (Figure 4a) that provides relevant copyright information ‘SUST’. Arnold scrambling algorithm [39] is used to encrypt it to improve security. The scrambling period of the watermark image is 48 times when all parameters in the Arnold formula are set to 1 (See Reference [39] for the specific formula of the Arnold scrambling algorithm). Figure 5 shows the original watermark image and the status of the image at the 15th, 25th, 35th, and 48th perturbations, where the perturbed watermark image at the 15th, 25th, and 35th perturbations is similar to a random noise map that is difficult to identify and has good concealment and reverts to the original state at the 48th perturbation. We chose the 15th disrupted image as the encrypted state’s watermark information and obtained its binary watermark sequence for the construction of zero watermarks.

#### 4.1.2. Zero Watermark Construction

The proposed watermarking scheme was performed using Python 3.10.4, Intel(R) Core(TM) i9-10900 CPU @ 2.80GHz, memory 32GB. Vector maps of buildings (Figure 5a), roads (Figure 5b), and water bodies (Figure 5c) were chosen as experimental data to test the dependability of the proposed watermarking scheme. Table 1 displays the detailed data information. The buildings, road, and water body maps each have 2708, 5115, and 844 elements, as well as 61,772, 86,367, and 422,764 vertices. The three maps generate 15,381, 21,237, and 15,294 feature points after Douglas–Peucker compression (all compression thresholds are set to 50 m), with compression ratios of 75.1 percent, 75.4 percent, and 96.4 percent, respectively. Point constraint blocking is performed on this basis. The initial block area parameter set is 1/4 of the entire area of the original data, and the coordinate point threshold is 5000. Finally, the building data yields 23 sub-blocks and zero watermarks, the road data yields 31 sub-blocks and zero watermarks, and the water map yields 22 sub-blocks and zero watermarks.

### 4.2. Robustness Evaluation

#### 4.2.1. Geometric Attacks

Translation, rotation, and scaling are examples of common geometric attacks. Watermark information is extracted from each sub-block of different data after various types and degrees of geometric attacks are performed on the experimental data. Table 2 lists the watermark images with the highest recognition degree extracted from different experimental data as well as different attack modes. It is evident that a watermark image with easily discernible copyright content can be extracted under all three geometric attack modes. Under both rotation and translation attacks, the extracted watermark images have NC values of 1, while under scaling attacks, the lowest NC value is 0.9821, and the highest is 1. Because of this, the proposed watermarking scheme can withstand attacks that use geometry. Because the CDTN architecture is both unique and invariant under rotation and translation attacks, the extracted spatial information remains unchanged after the DFT is calculated and remains consistent over time. In contrast, the experimental data produce a large change under the scaling attack, which has some effect on the feature points extraction. However, the CDTN possesses the characteristics of scaling invariance and overall invariance of local changes; the phase of DFT possesses scaling invariance; and, under the double insurance, the watermarking scheme also has strong robustness against the scaling attack.

#### 4.2.2. Cropping Attacks

The three sets of experimental data are attacked with different degrees of cropping. The cropping scale is about 20%, 25%, 50%, and 75% of the original area of the map. Based on this, the watermark images are extracted and restored, and the number of watermark images that can be restored in each set of data is counted. Table 3 displays the results, showing that all three data sets preserve a substantial number of complete sub-blocks and can extract a full watermark image at the 20%, 25%, and 50% crop stages, respectively. When the cropping scale hits 75%, there are just a handful of sub-blocks left from which to extract the watermark, whereas data (a) keeps the entire sub-block and extracts the watermark in its entirety so that it can be restored in full. Data (b) and data (c) both have NC values above 0.95 of extracted watermark images, indicating that the watermark images are recognized with a high degree of accuracy, despite the fact that a fully restored watermark image is not possible in these cases. Based on the experimental results, it is clear that the watermarking algorithm proposed in this paper is highly resistant to cropping attack. This excellent performance is mainly attributable to the strategy of blocking and watermarking supplementary bits in the data preprocessing stage, which builds multiple zero watermarks and can effectively resist a large degree of cropping attacks.

#### 4.2.3. Points Attacks

**Figure 6:**
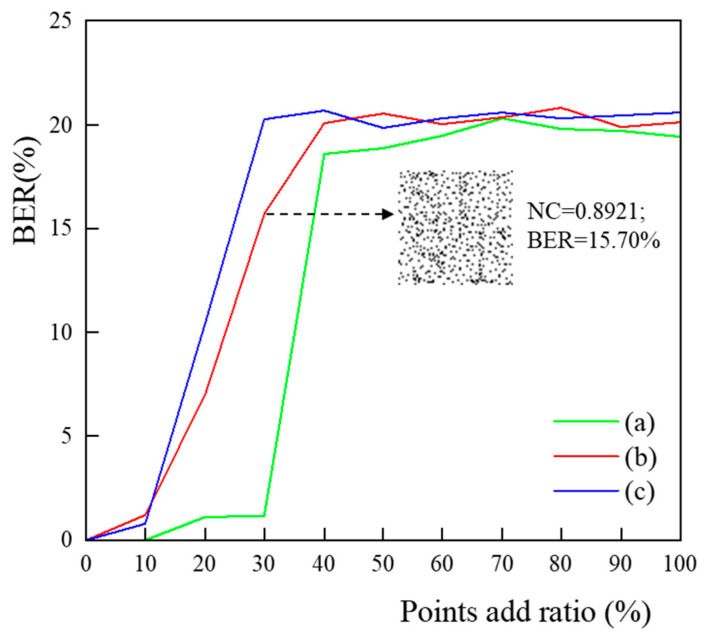
Result of points adding attack.

The coordinate point attack consists of three parts: adding or removing points at random and data compression. As the experiment progresses, we subject the experimental data to a range of data adding, deletions, and compression, all the while trying to extract the optimal watermark images and recording the resulting BER values. The points-adding attack is to add random coordinate points to the line elements of the experimental data, with the intensity of the incremental points increasing gradually by 10% of the number of vertices in the original experimental data, up to double the original data. The experimental outcomes are illustrated in Figure 6. At 20% strength of the points-adding attack, watermark images with low BER values can typically be retrieved from the data, and we believe that copyright identification is attained. When the attack intensity exceeds 20% (data (c) is larger than 30%), the BER values of the retrieved watermark image are typically greater than 15%, making it difficult to recognize legitimate copyright content and impossible to validate copyright. The points-adding attack affects feature point extraction, which in turn contributes to CDTN’s shaky building blocks. Experimental results show that the proposed zero-watermarking approach is still usable under the incremental point attack with an intensity level of up to 20%. The points-adding attack influences feature point extraction, which in turn leads to inconsistencies between the original and rebuilt CDTN. Experiments show that the proposed zero-watermarking scheme is reliable even when facing point-adding attacks with an intensity of 20% or less.

**Figure 7:**
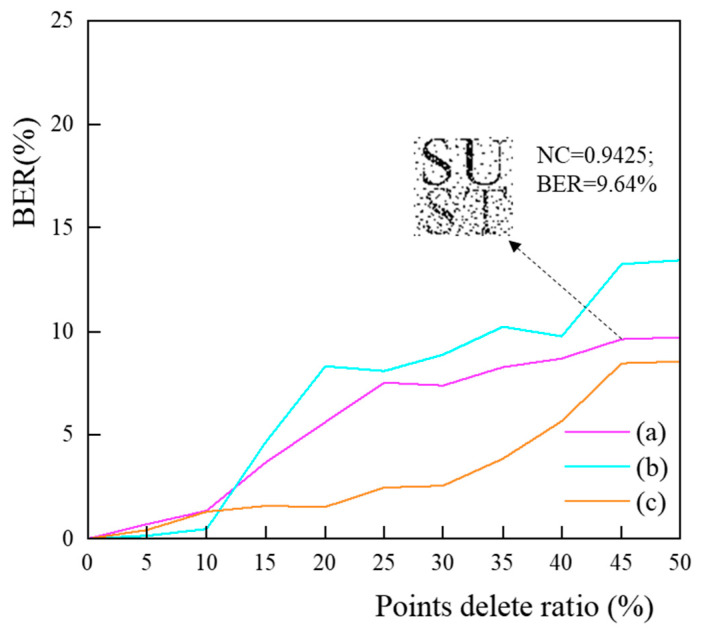
Result of points deletion attack.

The points-deletion attack is to randomly delete the vertices in the line elements, and the intensity of the deletion increases in the order of 5% of the number of vertices in the original data, up to 50% of the total number of vertices. Figure 7 displays the statistical outcomes of the points-deletion attacks. When the attack intensity is less than 50%, the BER values of the restored watermark images in different data are generally less than 10%. For example, in data (a) under a 45% density of points-deletion attack, the BER value of the extracted watermark image is 9.63%, and the NC value is 0.9425, allowing for clear identification of the copyright content. The results of the experiments show that the proposed watermarking scheme is highly resistant to the points-deletion attack. This is primarily because of the consistency of the feature points and the fact that local changes to the CDTN do not affect the whole. Furthermore, the majority of the deleted points are non-feature points, which have less of an effect on the reconstruction of the CDTN within some sub-blocks.

**Figure 8:**
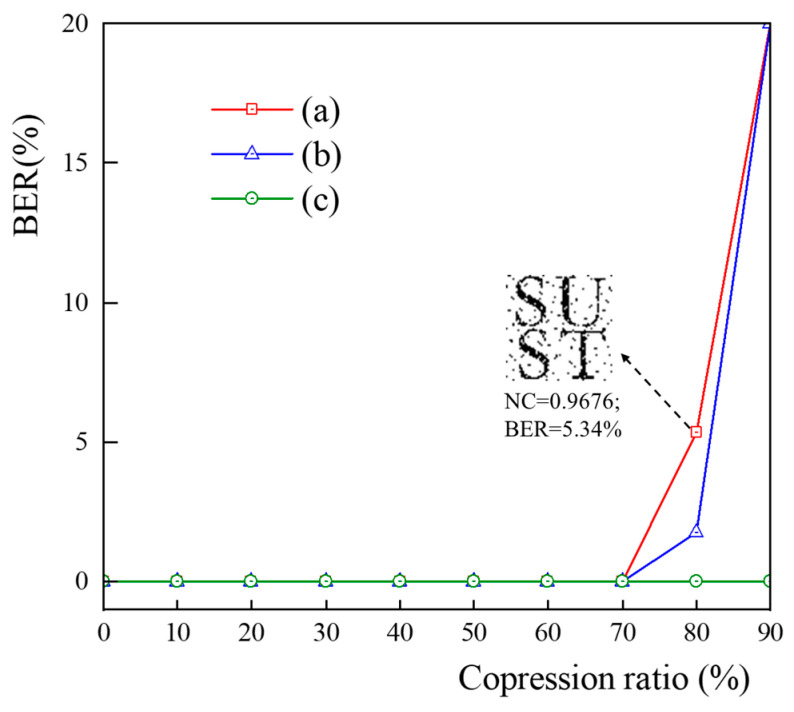
Result of compression attacks.

Data compression is a unique form of point attack that simplifies the vector map’s line elements. Figure 8 displays the BER values of the ideal watermark pictures that may be retrieved from three sets of experimental data at varying degrees of Douglas–Peucker compression. Evidently, it is always possible to extract watermark pictures with a BER of 0 as long as the compression ratio during data preprocessing is not exceeded. Additionally, even if the compression attack surpasses its original compression ratio by a small proportion, it can still be resisted well and remain robust. As depicted in Figure 8, when the compression ratio hits 80%, which slightly exceeds the predetermined compression ratio of 75% for data (a) and data (b), watermark images with high recognition can still be spotted. Since the proposed zero-watermarking is constructed based on feature points and the compression ratio is set to a high value, it retains a high level of robustness despite a relatively heavy compression attack.

#### 4.2.4. Coordinate System Transformation Attacks

When working with vector maps, coordinate system transformation is a common data editing technique that is also a highly specialized form of data processing due to its inherent geographical characteristics. Coordinate system transformation is one of the most common data editing methods encountered in the use of vector maps, and it is also a very specialized way of data processing with geographical characteristics. Vector map coordinate systems are broadly classified as geodetic and projection coordinate systems, with the WGS1984-UTM projection coordinate system being used for the experimental data in this paper. The effectiveness of the proposed watermarking is tested by converting each set of experimental data into a different geodetic and projection coordinate system, then extracting the watermark images and tallying the number of watermarks that can be read from each set of data. During the experiments, the experimental data are transformed into the WGS1972 and WGS1984 geodetic coordinate systems, as well as the WGS1972 UTM Zone49 projection coordinate system. Additionally, the experimental data are modified from the original 49th sub-band to the 50th sub-band to test the robustness of the projection attack under malicious attack. In Table 4 below, we can see that under different transformations, highly discriminative watermark images can be extracted in all three sets of experimental data. For example, when shifting to the geodetic coordinate system of WGS1984 and two different transformations of the projection coordinate system, we can extract a complete watermark image with an NC value of 1. Although the recoverable watermark images are on the small side after transformation to the WGS1972 geodetic coordinate system, the NC values are maintained at or above 0.97 in every case. Together, the coordinate system transformation robustness of the watermarking algorithm proposed is very high.

### 4.3. Comparative Analysis

Table 5 displays the comparative findings between the proposed watermarking scheme and the zero-watermarking schemes for vector maps that have proven strong robustness against multiple attacks in recent years. Among them, the reference [34] is our previous similar study, which agrees with the construction of CDTN but extracts different feature information. In the table, ‘√’ means that retrieving a valid watermarked image is possible, while ‘×’ means that doing so is impossible. In comparison to these representative studies, the present watermarking is only incapable of extracting the watermark image in larger scale points-adding attacks, whereas it is capable of extracting a valid watermark image in other attack patterns of varying degrees, demonstrating stronger robustness. In particular, most watermarking algorithms, such as [31,32,40], fail to extract watermark information in the face of larger-scale points-deletion and cropping attacks, while the proposed watermarking scheme shows a very strong robustness advantage. Due to the stability of the feature points and the geometric invariance of the CDTN, the proposed watermarking scheme can extract and verify the watermark with a small quantity of data, and this is also the primary reason why the proposed watermarking can extract the watermark image under the condition of different forms of coordinate points reduction. The algorithm described in [34] has a similar theoretical foundation to the proposed technique, notably with respect to the use of CDTN to extract feature information, and hence exhibits a very similar robustness performance in the comparison findings. However, Ref. [34] is unable to withstand the transformation of the projection coordinate system, whereas the technique presented in this study has been enhanced in this regard. The algorithm in [23] also mines the spatial angle values of vector maps as feature information based on feature points, which can complete the construction of zero watermark with a small amount of data. The results show that the method has very comprehensive robustness, especially in terms of coordinate point attacks, which is better than the algorithm in this paper. However, the scheme is difficult to resist coordinate system changes, while the proposed algorithm is greater in this regard. In general, the proposed zero-watermarking algorithm offers a more comprehensive performance in terms of robustness than the majority of current studies.

## 5. Conclusions

Based on mining the geometric statistical information in the spatial domain of vector maps and employing the geometric invariance of the frequency domain transformation coefficients, a robust zero-watermarking scheme for vector maps is presented in this study. In spatial domain information mining, feature points are used to build a CDTN, and the angle values in the CDTN are used to obtain the initial set of features. The extracted angle value sequence is changed using DFT, and the zero watermarks are made using the binary conversion of the phase sequence of DFT as the feature information. The uniqueness of CDTN, the fact that local alterations have no effect globally, and the geometric invariance of DFT transform coefficients provide a solid foundation of robustness for the proposed zero-watermarking scheme. Experimental results demonstrate that the proposed watermarking scheme has good performance against common geometric attacks, clipping attacks, and point attacks, making it a potential technical reference solution for copyright protection of vector maps. The proposed algorithm needs computational efficiency improvements. Zero-watermark construction takes roughly half an hour for data (b) with little information and around 20 h for data (c) with much information, which affects the algorithm’s practicability. In future work, we will concentrate on the computational efficiency and interpretive threats in the zero-watermarking technique for vector maps. For instance, we may choose a language that runs faster than Python or simplifies code and combine the blockchain method to ensure that the zero-watermark key and protected data can be traced, hence enhancing the algorithm’s practical utility.

## Figures and Tables

**Table 1:** Information of original and preprocessed experimental data.

Data Set	Number of Features	Number of Points	Number of Feature Points	Number of Blocks	Number of Zero Watermarks
Buildings	2708	61,772	15,381	23	23
Roads	5115	86,367	21,237	31	31
Rivers	844	422,764	15,294	22	22

**Table 2:** Results of geometric attacks.

Attack Mode	Scale	*W′*/(a)	*W′*/(b)	*W′*/(c)
Rotation	30°	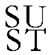	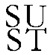	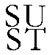
		NC = 1, BER = 0%	NC = 1, BER = 0%	NC = 1, BER = 0%
	90°	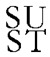	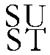	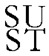
		NC = 1, BER = 0%	NC = 1, BER = 0%	NC = 1, BER = 0%
	180°	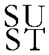	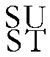	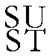
		NC = 1, BER = 0%	NC = 1, BER = 0%	NC = 1, BER = 0%
Scaling	0.5 times	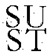	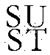	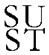
		NC = 0.9976, BER = 0.039%	NC = 0.9973, BER = 0.044%	NC = 1, BER = 0%
	2 times	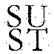	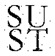	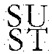
		NC = 0.9957, BER = 0.071%	NC = 0.9954, BER = 0.076%	NC = 0.9960, BER = 0.066%
	5 times	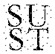	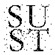	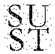
		NC = 0.9840, BER = 2.64%	NC = 0.9821, BER = 2.95%	NC = 0.9834, BER = 2.73%
Translation	10 m	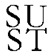	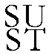	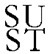
		NC = 1, BER = 0%	NC = 1, BER = 0%	NC = 1, BER = 0%
	20 m	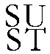	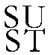	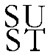
		NC = 1, BER = 0%	NC = 1, BER = 0%	NC = 1, BER = 0%
	50 m	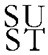	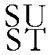	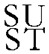
		NC = 1, BER = 0%	NC = 1, BER = 0%	NC = 1, BER = 0%

**Table 3:** Results of cropping attacks.

Crop Scale	*W′*/(a)	*W′* Count/(a)	*W′*/(b)	*W′* Count/(b)	*W′*/(c)	*W′* Count/(c)
20%	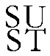	15	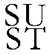	28	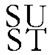	19
	NC = 1, BER = 0%		NC = 1, BER = 0%		NC = 1, BER = 0%	
25%	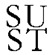	16	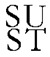	23	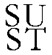	16
	NC = 1, BER = 0%		NC = 1, BER = 0%		NC = 1, BER = 0%	
50%	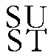	6	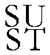	17	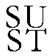	8
	NC = 1, BER = 0%		NC = 1, BER = 0%		NC = 1, BER = 0%	
75%	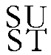	1	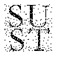	3	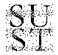	1
	NC = 1, BER = 0%		NC = 0.9577, BER = 6.98%		NC = 0.9522, BER = 7.91%	

**Table 4:** Results of coordinate system transformation attacks.

Attack Mode		*NC_max_*/ (a)	*W′* Count/(a)	*NC_max_*/ (b)	*W′* Count/(b)	*NC_max_*/ (c)	*W′* Count/(c)
Geodetic coordinate system transformation	WGS1972	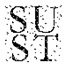	5	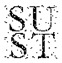	8	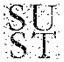	5
		NC = 0.9743, BER = 4.20%		NC = 0.9729, BER = 4.49%		NC = 0.9744, BER = 3.91%	
	WGS1984	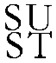	23	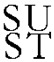	31	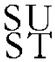	22
		NC = 1 BER = 0%		NC = 1 BER = 0%		NC = 1 BER = 0%	
Projection coordinate system transformation	WGS1972_UTM_Zone49	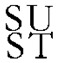	23	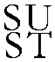	31	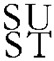	22
		NC = 1 BER = 0%		NC = 1 BER = 0%		NC = 1 BER = 0%	
	WGS1984_UTM_Zone50	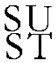	18	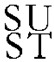	19	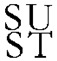	13
		NC = 1 BER = 0%		NC = 1 BER = 0%		NC = 1 BER = 0%	

**Table 5:** Results of comparative analysis.

Attack Mode	Scale	Reference [31]	Reference [32]	Reference [23]	Reference [34]	Reference [40]	The Proposed
Rotation	30°	√	×	√	√	×	√
	90°	√	×	√	√	×	√
	180°	√	×	√	√	×	√
Scaling	0.5 times	√	√	√	√	√	√
	2 times	√	√	√	√	√	√
	5 times	√	√	√	√	√	√
Translation	10 m	√	√	√	√	√	√
	20 m	√	√	√	√	√	√
	50 m	√	√	√	√	√	√
Cropping	20%	√	√	√	√	√	√
	25%	×	√	√	√	×	√
	50%	×	×	√	√	×	√
	75%	×	×	√	√	×	√
Points adding	25%	√	×	√	√	√	√
	50%	√	×	√	×	√	×
	100%	√	×	√	×	×	×
Points deletion	2%	√	×	√	√	√	√
	10%	×	×	√	√	×	√
	20%	×	×	√	√	×	√
Compression	25%	√	×	√	√	√	√
	50%	√	×	√	√	√	√
	75%	√	×	√	√	×	√
Coordinate system transform	Geodetic coordinate system	√	√	×	√	√	√
	Projection coordinate system	√	√	×	×	√	√

## Data Availability

The data used in this study can be accessible by request to the corresponding author.

## References

[1] Usman M., Jan M.A., He X., Chen J. (2019). A survey on big multimedia data processing and management in smart cities. ACM Comput. Surv..

[2] Abubahia A., Cocea M. (2018). Evaluating the topological quality of watermarked vector maps. Appl. Soft. Comput..

[3] Xu X., Zhang X. (2022). Reversible watermarking for vector maps based on interval mapping and maximum perturbation region. J. Geod. Geoinf. Sci..

[4] Pham G.N., Ngo S.T., Bui A.N., Tran D.V., Lee S.H., Kwon K.R. (2019). Vector map random encryption algorithm based on multi-scale simplification and Gaussian distribution. Appl. Sci..

[5] Wang X., Shao C., Xu X., Niu X. (2007). Reversible data-hiding scheme for 2-D vector maps based on difference expansion. IEEE Trans. Inf. Forensics Secur..

[6] Jang B.J., Lee S.H., Kwon K.R. (2014). Perceptual encryption with compression for secure vector map data processing. Digit. Signal Process..

[7] Serra-Ruiz J., Qureshi A., Megías D. (2019). Entropy-based semi-fragile watermarking of remote sensing images in the wavelet domain. Entropy.

[8] Wang N., Men C. (2012). Reversible fragile watermarking for 2-D vector map authentication with localization. Comput. Aided Design.

[9] Zhu C. (2017). Research progresses in digital watermarking and encryption control for geographical data. J. Geod. Geoinf. Sci..

[10] Yang Z., Sun Q., Qi Y., Li S., Ren F. (2022). A Hyper-Chaotically Encrypted Robust Digital Image Watermarking Method with Large Capacity Using Compress Sensing on a Hybrid Domain. Entropy.

[11] Cedillo-Hernandez M., Cedillo-Hernandez A., Nakano-Miyatake M., Perez-Meana H. (2020). Improving the management of medical imaging by using robust and secure dual watermarking. Biomed. Signal. Proces..

[12] Wang C., Wang X., Xia Z., Zhang C. (2019). Ternary radial harmonic Fourier moments based robust stereo image zero-watermarking algorithm. Inform. Sci..

[13] Wang B., Shi J., Wang W., Zhao P. (2022). Image copyright protection based on blockchain and zero-watermark. IEEE Trans. Netw. Sci. Eng..

[14] Cox G.S., Jager G. A Survey of Point Pattern Matching Techniques and a New Approach to Point Pattern Recognition. Proceedings of the 1992 South African Symposium on Communications and Signal Processing.

[15] Wang C., Peng Z., Peng Y., Yu L., Wang J., Zhao Q. (2012). Watermarking geographical data on spatial topological relations. Multimed. Tools Appl..

[16] Tong D., Zhu C., Ren N. (2018). Watermarking algorithm applying to small amount of vector geographical data. Acta Geod. Cartogr. Sin..

[17] Lee S.H., Kwon K.R. (2013). Vector watermarking scheme for GIS vector map management. Multimed. Tools Appl..

[18] Peng Z., Yue M., Wu X., Peng Y. (2015). Blind watermarking scheme for polylines in vector geo-spatial data. Multimed. Tools Appl..

[19] Abubahia A., Cocea M. (2017). Advancements in GIS map copyright protection schemes—A critical review. Multimed. Tools Appl..

[20] Zhu C., Ren N., Xu D. (2022). Geo-information security technology: Progress and prospects. Acta Geod. Cartogr. Sin..

[21] Ren N., Zhao M., Zhu C., Sun X., Zhao Y. (2021). Commutative encryption and watermarking based on SVD for secure GIS vector data. Earth Sci. Inform..

[22] Xi X., Zhang X., Sun Y., Jiang X., Xin Q. (2020). Topology-preserving and geometric feature-correction watermarking of vector maps. IEEE Access.

[23] Ren N., Zhao Y., Zhu C., Zhou Q., Xu D. (2021). Copyright protection based on zero watermarking and blockchain for vector maps. ISPRS Int. J. Geo-Inf..

[24] Zhou Q., Zhu C., Ren N., Chen W., Gong W. (2021). Zero watermarking algorithm for vector geographic data based on the number of neighboring features. Symmetry.

[25] Wang S., Zhang L., Zhang Q., Li Y. (2023). A zero-watermarking algorithm for vector geographic data based on feature invariants. Earth Sci. Inform..

[26] Wen Q., Sun T., Wang S. (2003). Concept and Application of Zero-Watermark. Acta Electron. Sin..

[27] Li A., Lin B., Chen Y., Lv G. (2008). Study on copyright authentication of GIS vector data based on zero-watermarking. Int. Arch. Photogramm. Remote Sens. Spat. Inf. Sci..

[28] Sun Y., Li D. (2017). Vector map zero watermark based on node feature. Geog. Geo-inf. Sci..

[29] Wang X., Huang D., Zhang Z. (2012). A robust zero-watermarking algorithm for vector digital maps based on statistical characteristics. J. Softw..

[30] Peng Y., Yue M. (2015). A zero-watermarking scheme for vector map based on feature vertex distance ratio. J. Electr. Comput. Eng..

[31] Lv W., Zhang L. (2018). A DFT based zero-watermarking algorithm for vector geodata. J. Geomatics Sci. Technol..

[32] Li W., Yan H., Wang Z., Zhang L., Lu X. (2017). A zero-watermarking algorithm for vector geo-spatial data based on logistic chaotic mapping and DFT. Sci. Surv. Mapp..

[33] Han Z. (2017). Study on Lossless Digital Watermarking Algorithm for Vector Map Based on Space Feature.

[34] Xu X., Zhang X., Liang W., Xin Q., Zhang P. (2019). Dual zero-watermarking scheme for two-dimensional vector map based on Delaunay triangle mesh and singular value decomposition. Appl. Sci..

[35] Ren N., Wu W., Zhu C. (2015). An accurate authentication algorithm based on point constraint block for vector geographic data. J. Geo-Inf. Sci..

[36] Rippa S. (1990). Minimal roughness property of the Delaunay triangulation. Comput. Aided Geom. Des..

[37] Huber S., Held M., Meerwald P., Kwitt R. (2014). Topology-preserving watermarking of vector graphics. Int. J. Comput. Geom. Appl..

[38] Urvoy M., Goudia D., Autrusseau F. (2014). Perceptual DFT watermarking with improved detection and robustness to geometrical distortions. IEEE Trans. Inf. Forensics Secur..

[39] Arnold V.I., Avez A. (1968). Ergodic Problems of Classical Mechanics.

[40] Li W., Yan H., Wang Z., Zhang L. (2016). A zero-watermarking algorithm for vector linear feature data. J. Geom. Sci. Technol..

